# Fe_3_O_4_/SiO_2_ decorated trimesic acid-melamine nanocomposite: a reusable supramolecular organocatalyst for efficient multicomponent synthesis of imidazole derivatives

**DOI:** 10.1038/s41598-023-27408-7

**Published:** 2023-01-09

**Authors:** Babak Fattahi, Mohammad G. Dekamin

**Affiliations:** Pharmaceutical and Heterocyclic Compounds Research Laboratory, Department of Chemistry, Iran University of Science and Technology, Tehran, 16846-13114 Iran

**Keywords:** Environmental chemistry, Drug discovery, Chemistry, Catalyst synthesis, Heterogeneous catalysis, Organocatalysis

## Abstract

This article describes supramolecular Fe_3_O_4_/SiO_2_ decorated trimesic acid-melamine (Fe_3_O_4_/SiO_2_-TMA-Me) nanocomposite that can be prepared with features that combine properties of different materials to fabricate a structurally unique hybrid material. In particular, we have focused on design, synthesis and evaluation a heterogeneous magnetic organocatalyst containing acidic functional-groups for the synthesis of biologically important imidazole derivatives in good to excellent yields. The introduced Fe_3_O_4_/SiO_2_-TMA-Me nanomaterial was characterized by different techniques such as FTIR, XRD, EDX, FESEM, TEM, TGA and DTA. As a noteworthy point, the magnetic catalytic system can be recycled and reused for more than seven consecutive runs while its high catalytic activity remains under the optimized conditions.

## Introduction

The total synthetic approaches or even single-step reactions are being adjusted to the basic principles of green and sustainable chemistry that purpose to reduce the production of hazardous materials under various reaction conditions^1–3^. The designed-procedures for the preparation of nanomaterials including magnetic nanoparticles and their catalytic activities are entirely verified in the field of greener and atom-economic reactions especially multicomponent reactions^4–7^. Indeed, the use of magnetic decorated organic structures is gaining significant attention in the field of catalysis for organic transformations, mainly due to opportunities in providing new structural diversities^8–13^. The design and construction of new structures is achieved with the aim of improving the structural characteristics and enhancing desired catalytic performance in the definite reactions. For instance, by using diverse acidic organic functional groups with different acid strengths in the catalytic systems structure, the acidic properties of the final composition can be tuned.

Supramolecular chemistry, based on distinct interactions between small molecules as well as polymers, is a great tool to achieve superior, self-assembled molecular structures with an increased level of complexity^14^. According to these interactions, it is possible to prepare pseudo-supramolecular structures through sequential and predictable bonds between different organic moieties^15^. Further, grafting of the functionalized organic chains to the magnetic substrates is one of the best procedures for the construction of heterogeneous catalysts^16^ with high stability, activity and reusability^17–22^.

**Figure 1:**
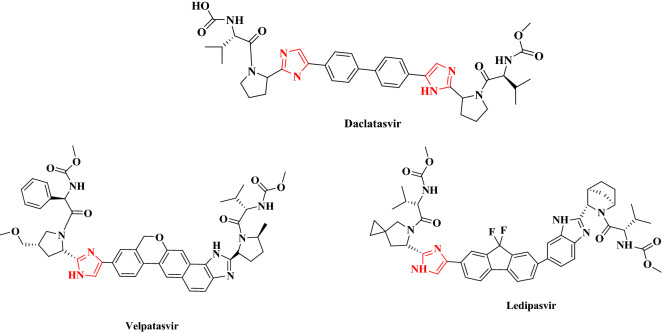
Examples of medicines containing the imidazole scaffold.

New supramolecular catalytic systems can promote the synthesis of fine chemicals through the multicomponent reaction (MCR) strategy very fast^23–26^. Definitely, heterocyclic scaffolds representing biological properties and medicinal applications including imidazole derivatives are an important category of such organic compunds^27^. Obviously, some drugs such as Daclatasvir (antiviral), Ledipasvir (antiviral), Velpatasvir (antiviral), Ketoconazole (antifungal), Clonidine (anti-hypertension), etc. have an imidazole core in their structures (Fig. 1).

**Figure 2:**
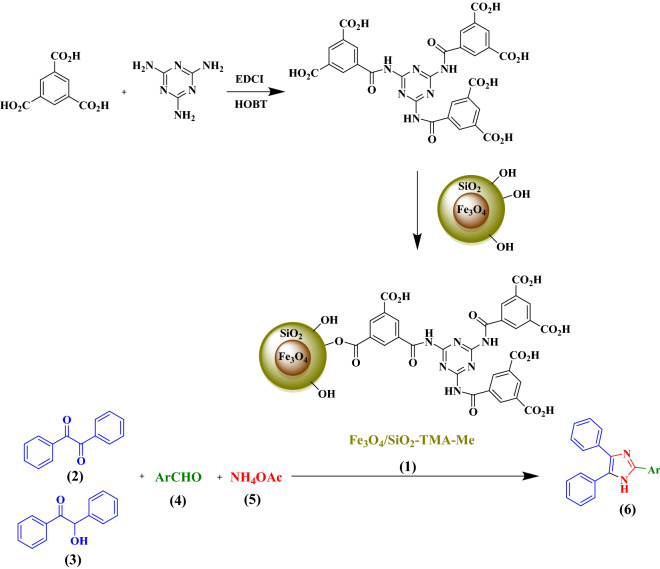
Schematic preparation of the Fe_3_O_4_/SiO_2_-TMA-Me nanocomposite (**1**) for the three-component condensation of benzil (**2**) or benzoin (**3**), aldehydes (**4**), and ammonium acetate (**5**) to afford imidazole derivatives **6**.

In continuation of our ongoing efforts to design heterogeneous catalysts for different MCRs^26–34^, we wish herein to introduce preparation and fully characterization of the new magnetic Fe_3_O_4_/SiO_2_ decorated trimesic acid-melamine nanocomposite (Fe_3_O_4_/SiO_2_-TMA-Me, **1**). Furthermore, its catalytic activity was investigated in the three-component synthesis of imidazole derivatives from benzil (**2**) or benzoin (**3**), aldehydes (**4**), and ammonium acetate (5, Fig. 2). To the best of our knowledge, there is not any report for the use of pseudo-supramolecular heterogeneous magnetic organocatalyst having acidic functional groups for the synthesis of imidazole derivatives.

## Results and discussion

### Characterization of the Fe_3_O_4_/SiO_2_-TMA-Me (1)

**Figure 3:**
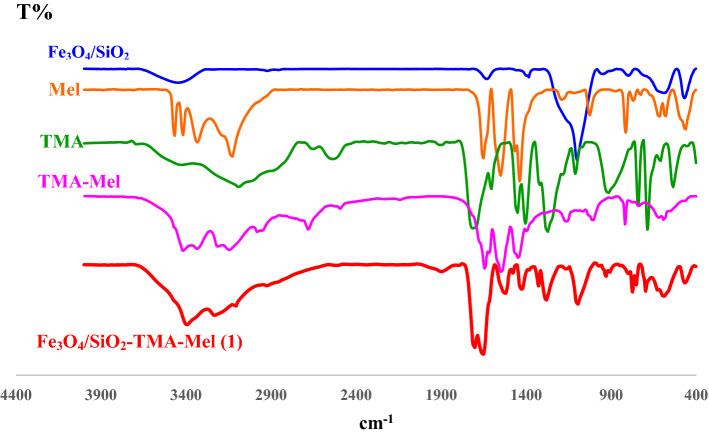
FTIR spectra of the Fe_3_O_4_/SiO_2_, melamine (Mel), trimesic acid (TMA), melamine-trimesic acid amide (Mel-TMA) and Fe_3_O_4_/SiO_2_-TMA-Me solid acid (**1**).

The as prepared Fe_3_O_4_/SiO_2_-TMA-and energy dispersive X-ray (EDX)Me nanomaterial (**1**) was characterized using various analytical techniques and methods such as Fourier transform infrared (FTIR) and energy dispersive X-ray (EDX) spectroscopy, field emission scanning electron microscopy (FESEM), transmission electron microscopy (TEM), X-ray diffraction (XRD), thermogravimetric (TGA), and differential thermal (DTA) analysis. The FTIR spectra of Fe_3_O_4_/SiO_2_, melamine (Mel) trimesic acid (TMA), melamine-trimesic acid amide (Mel-TMA) and Fe_3_O_4_/SiO_2_-TMA-Me solid acid (**1**) are show in Fig. 3.

As shown in Fig. 3, the Fe_3_O_4_/SiO_2_-TMA-Mel solid acid (**1**) presented a very strong and broad band, covering a wide range between 2800 and 3600 cm^-1^, for the O–H stretching vibrations of the carboxylic acid functional groups as well as Fe_3_O_4_/SiO_2_. Furthermore, the signals at 1730, 1710 and 1683 cm^−1^ are assigned to the carbonyl groups of ester, acid and amide, respectively. It should be noted that the presence of carbonyl group of ester indicates the formation of a covalent bond between the acid groups of trimesic acid and the magnetic core/shell. Furthermore, the asymmetric vibration signals of Si–O–Si and Si–OH as well as the symmetric vibration signal of Si–O–Si could be seen at 1090, 930 and 790 cm^–1^. In addition, the characteristic band for Fe–O stretching vibrations was observed at 560 cm^−1^.

**Figure 4:**
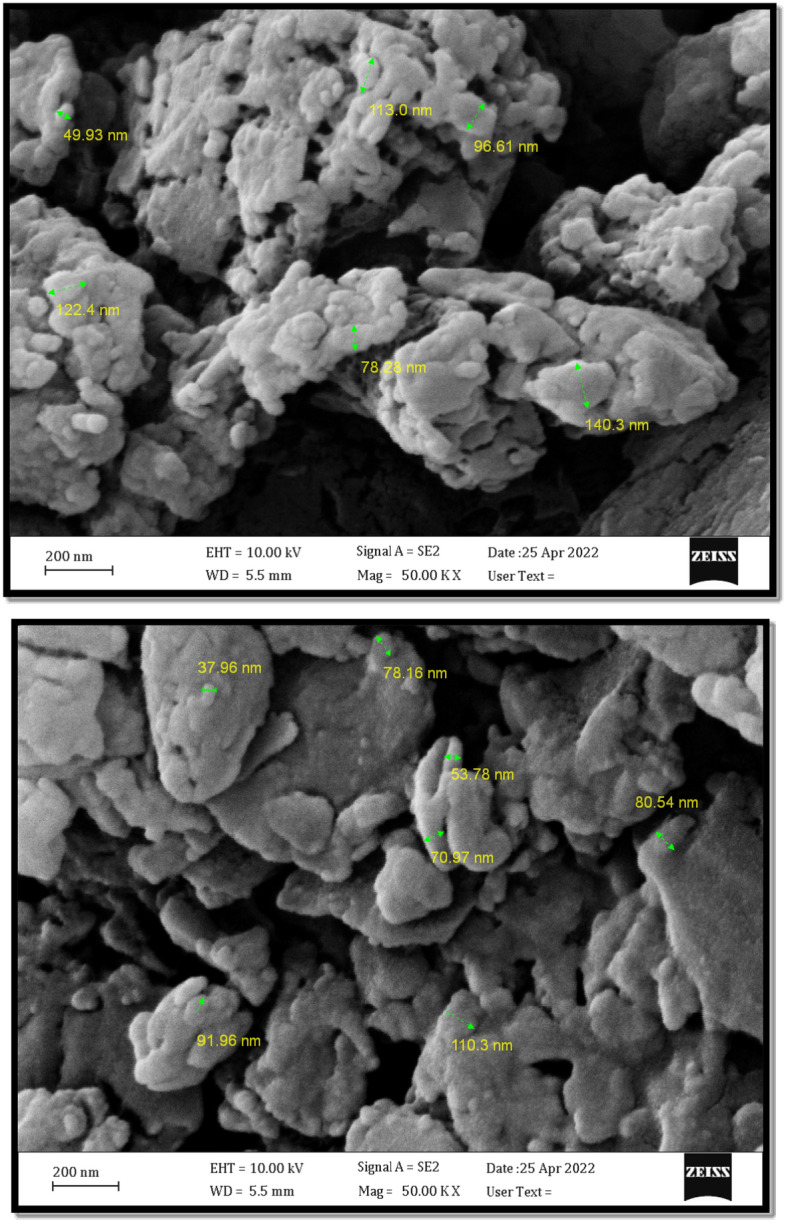
FESEM images of the Fe_3_O_4_/SiO_2_-TMA-Me nanocomposite (**1**).

**Figure 5:**
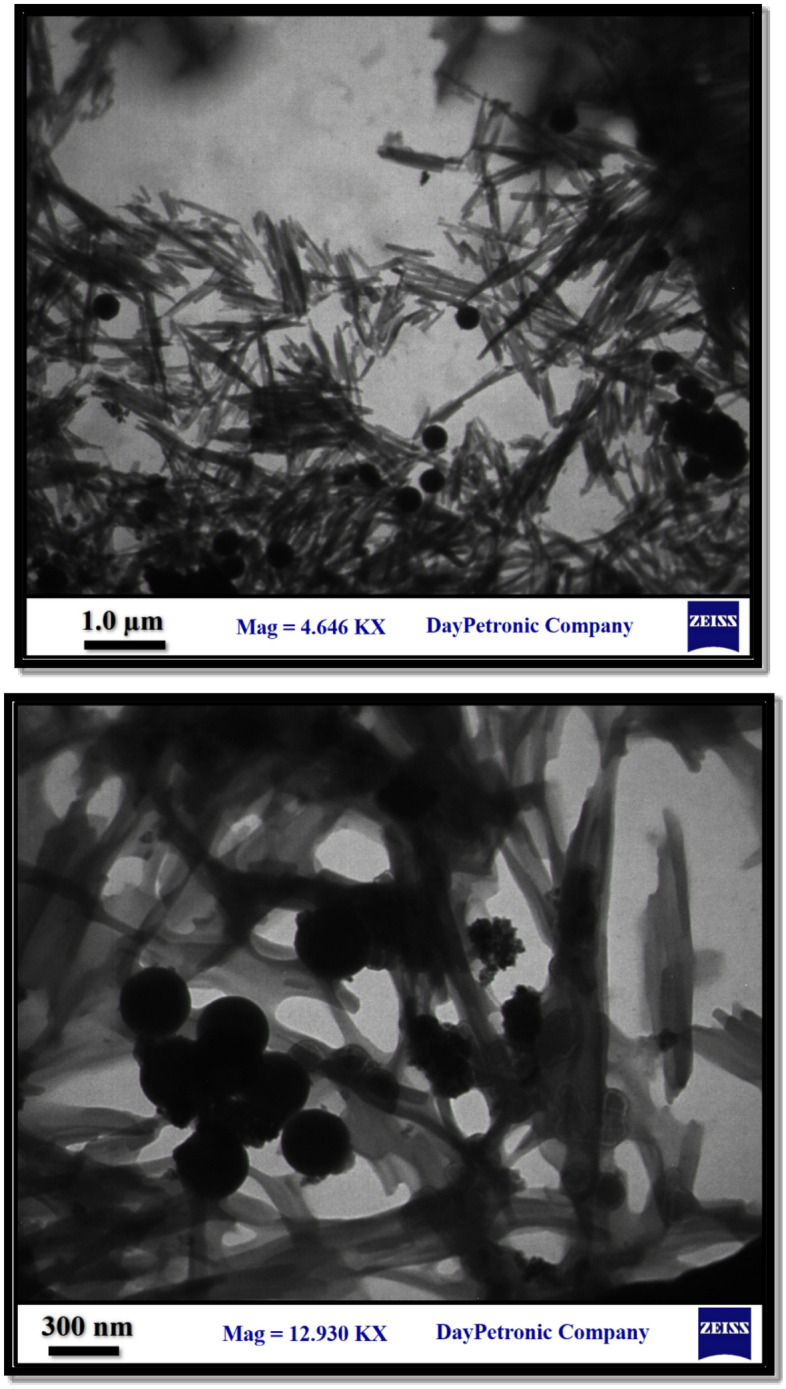
TEM images of the Fe_3_O_4_/SiO_2_-TMA-Me nanomaterial (**1**) ﻿in 1.0 µm and 300 nm scales.

The morphological features and particles size of the new magnetic nanocomposite Fe_3_O_4_/SiO_2_-TMA-Me nanocomposite (**1**) were examined by FESEM and TEM experiments (Figs. 4 and 5). The catalyst nanoparticles are approximately spherical and have been distributed with an average diameter of about 75 nm. On the other hand, TEM images (**Fig. ****5**) obviously demonstrate decoration of core/shell magnetic nanoparticles on the trimesic acid/melamine rod-shaped structure. Also, the TEM images can be considered as a confirmation of the pseudo-supramolecular structure. Indeed, by considering this point that the Fe_3_O_4_/SiO_2_ is a core/shell structure, according to its preparation method, the rod-shaped particles shown in FESEM images may be attributed to the polymerized structure of melamine and trimesic acid.

**Figure 6:**
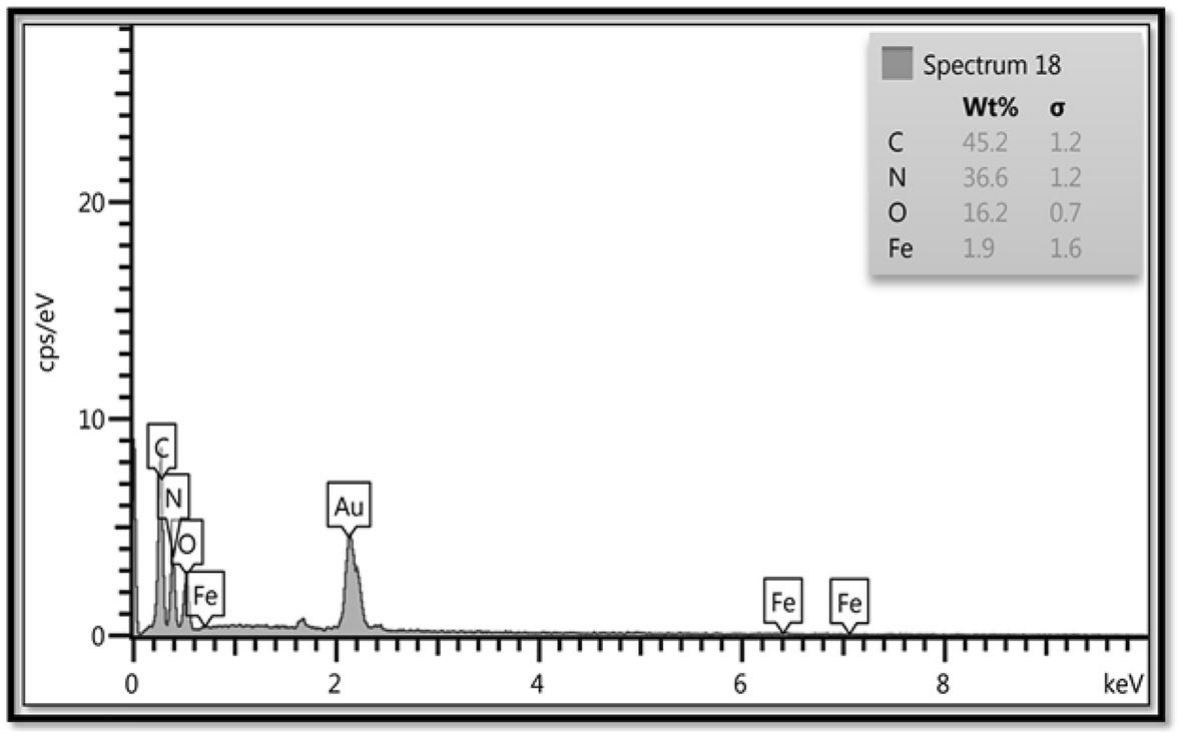
EDX spectrum of the Fe_3_O_4_/SiO_2_-TMA-Me nanocomposite (**1**).

The Energy dispersive spectroscopy (EDX) of the Fe_3_O_4_/SiO_2_-TMA-Me (**1**) is shown in Fig. 6. The EDX spectrum indicates that the introduced nanocatalyst **1** is composed of Fe, O, N and C elements.

**Figure 7:**
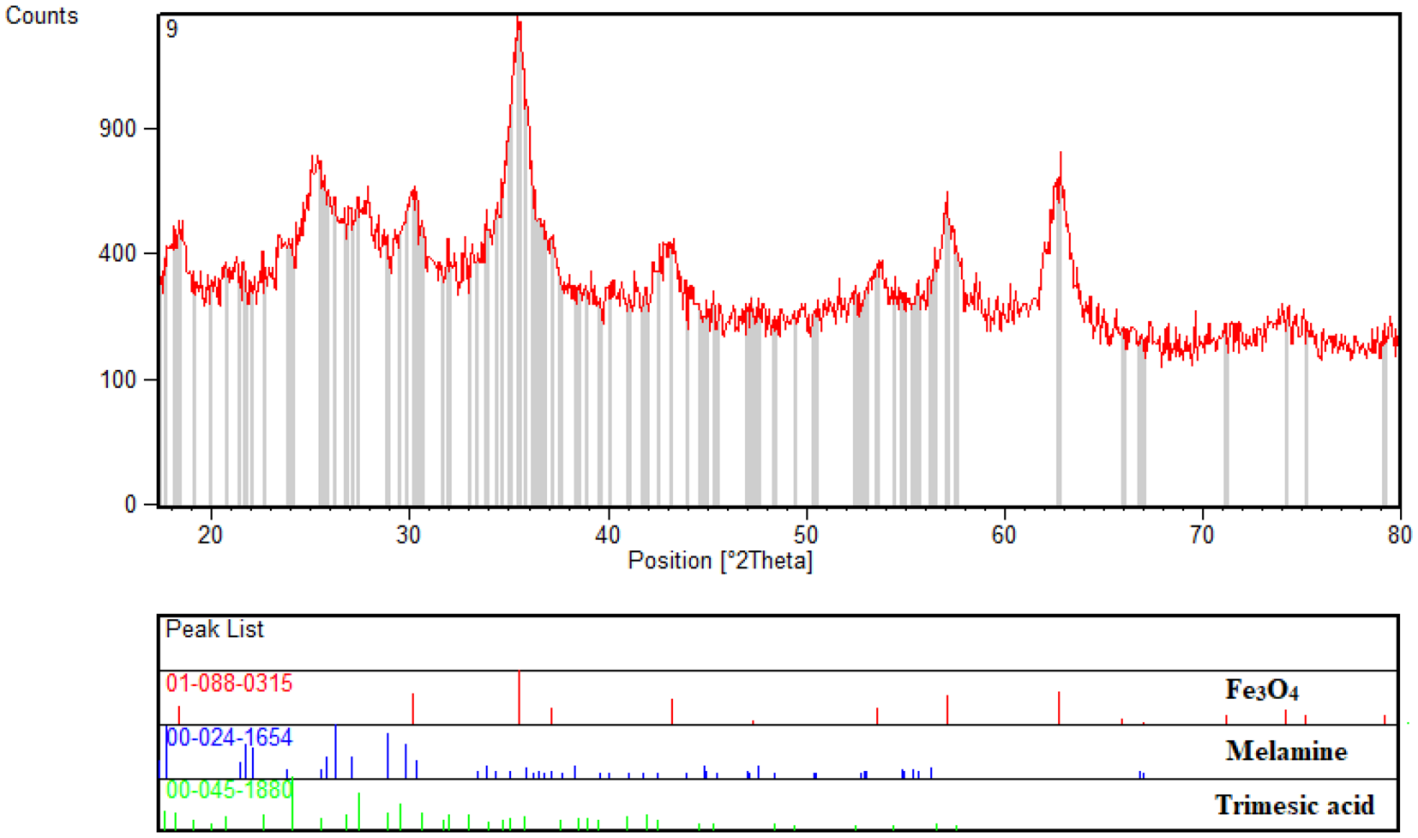
XRD pattern of the Fe_3_O_4_/SiO_2_-TMA-Me  nanocomposite (**1**).

Also, Fig. 7 shows the XRD pattern of the Fe_3_O_4_/SiO_2_-TMA-Me nanomaterial (**1**). The XRD patterns of both melamine and trimesic acid are also illustrated for comparison as offset patterns. The diffraction peaks at 2θ values of 30.20, 35.39, 36.89, 53.31, 56.98, 73.91° can be assigned to the reflections of cubic Fe_3_O_4_ (JCPDS No. 01–088-0315) On the other hand, the well-defined high intensity diffraction signals (2θ) at 13.41, 17.95, 21.65, 22.25, 26.28, 28.90 and 29.91° are in accordance with the monoclinic crystal system of melamine (JCPDS no. 024–1654). Also, other remaining diffraction peaks can be attributed to the reflections of trimesic acid according to the JCPDS No. 00–045-1880.

**Figure 8:**
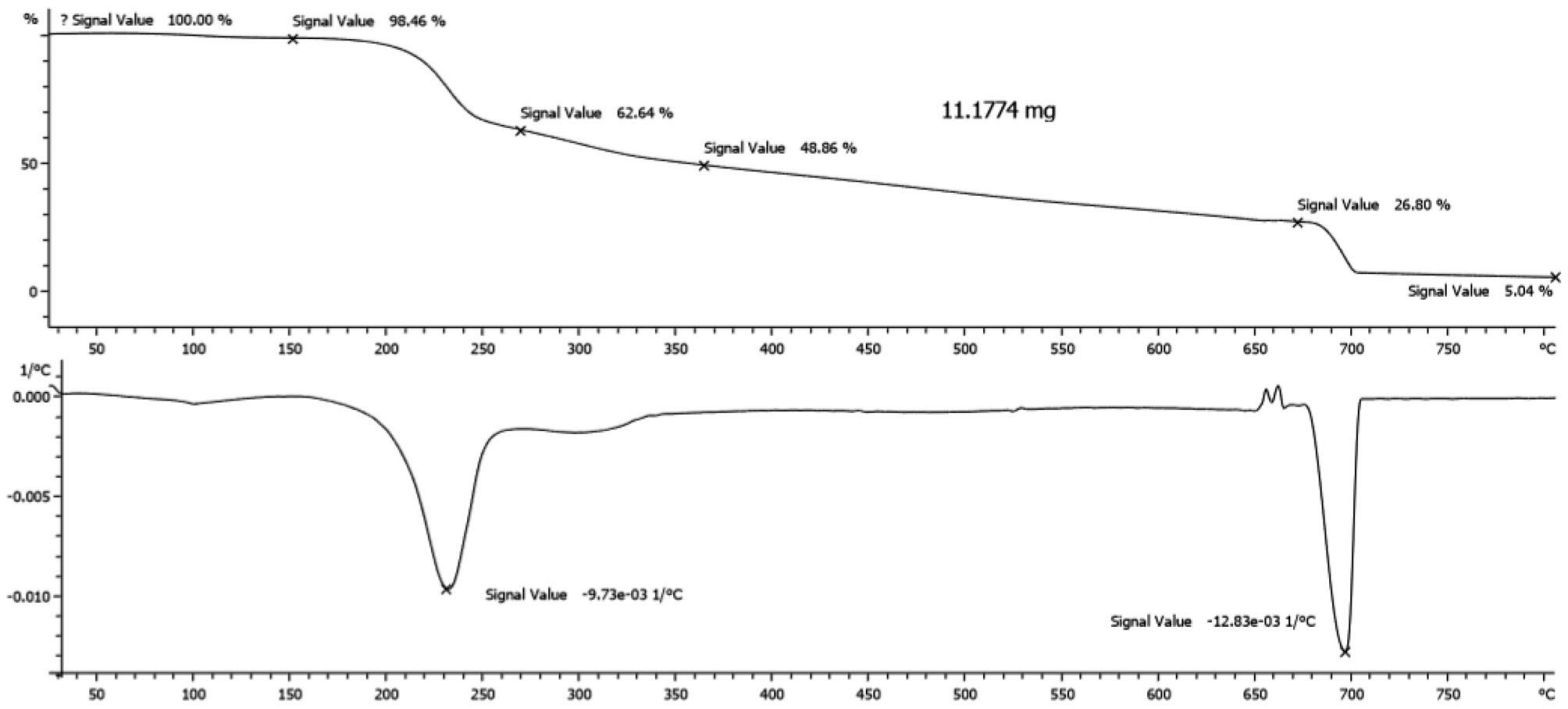
TGA/DTA curves of the Fe_3_O_4_/SiO_2_-TMA-Me, nanocomposite (**1**).

On the other hand, the TGA and DTA curves of the Fe_3_O_4_/SiO_2_-TMA-Me nanomaterial (**1**) in Fig. 8 show that the slight weight loss between 35—150 °C can be assigned to the elimination of adsorbed solvent or water molecules on its surface or trapped inside of the sample. Also, the weight losses between 150–270 °C and 270–370 °C is attributed to the partial or complete decomposition of trimesic acid moiety as well as condensation of the melamine units to melam through losing of NH_3_ molecules in the Fe_3_O_4_/SiO_2_-TMA-Me (**1**) structure. Moreover, the next weight loss can be interpreted by condensation of the silanols to siloxanes as well as forming more Fe −O− Fe bridges. The last step of weight loss between between 670 and 800 °C is due to complete decomposition of organic residue and remaining the inorganic Fe_3_O_4_/SiO_2_.

### Investigation of the catalytic activity of Fe_3_O_4_/SiO_2_-TMA-Me (1) for the synthesis of imidazole derivatives 6a–m

After characterization of the Fe_3_O_4_/SiO_2_ decorated trimesic acid-melamine (Fe_3_O_4_/SiO_2_-TMA-Mel) nanocomposite (**1**), the three-component synthesis of imidazole derivatives was chosen to examine its catalytic activity. For this purpose, the condensation of benzoin (**3**, 1 mmol), 4-chlorobenzaldehyde (**4a**, 1 mmol) and NH_4_OAc (**5**, 2.5 mmol) was selected as the model reaction for the synthesis of **6a**. The reactions were optimized considering various parameters such as solvent, catalyst loading and temperature. The results are reported in Table 1.

**Table 1:** Optimization of conditions in the model reaction of benzoin (**3**), 4-chlorobenzaldehyde (**4a**), ammonium acetate (**5**) under different conditions in the presence of the Fe_3_O_4_/SiO_2_-TMA-Me solid acid (**1**).^a^


Entry	Catalyst	Catalyst loading (mg)	Condition	Time (min)	Yield (%)^b^
1	–	–	Solvent-free/100 °C	300	34
2	–	–	H_2_O/r.t	300	Trace
3	–	–	EtOH/r.t	300	Trace
4	–	–	EtOH/reflux	300	40
5	Fe_3_O_4_/SiO_2_-TMA-Mel (**1**)	20	EtOH/r.t	120	60
6	Fe_3_O_4_/SiO_2_-TMA-Mel (**1**)	20	EtOH/60 °C	120	72
7	Fe_3_O_4_/SiO_2_-TMA-Mel (**1**)	20	CH_2_Cl_2_/r.t	180	58
8	Fe_3_O_4_/SiO_2_-TMA-Mel (**1**)	20	CH_3_CN/reflux	60	83
9	Fe_3_O_4_/SiO_2_-TMA-Mel (**1**)	20	EtOH/reflux	30	96
10	Fe_3_O_4_/SiO_2_-TMA-Mel (**1**)	15	EtOH/reflux	30	95
11	Fe_3_O_4_/SiO_2_-TMA-Mel (**1**)	10	EtOH/reflux	35	95
12	Fe_3_O_4_/SiO_2_-TMA-Mel (**1**)	5	EtOH/reflux	45	92
13	Fe_3_O_4_/SiO_2_-TMA-Mel (**1**)	2	EtOH/reflux	60	87
14	Melamine	10	EtOH/reflux	35	75
15	Trimesic acid	10	EtOH/reflux	35	79
16	Fe_3_O_4_/SiO_2_	10	EtOH/reflux	35	73
17	Trimesic acid-Melamine	10	EtOH/reflux	35	84

^a^Reaction conditions: benzoin (**3**, 1 mmol), 4-chlorobenzaldehyde (**4a**, 1 mmol), NH_4_OAc (**5**, 2.5 mmol), Fe_3_O_4_/SiO_2_-TMA-Me (**1**) and solvent (2 ml) unless otherwise noted.

^b^Isolated yield.

The results of using different conditions in model reaction have been presented in Table 1. It is noteworthy that a very low yield of the desired product **6a** was obtained in the absence of the Fe_3_O_4_/SiO_2_-TMA-Me (**1**) (Table 1, Entries 1–4). By using different solvents, the best result was obtained with ethanol at room temperature. In the next step, the amount of catalyst loading was optimized (Table 1, Entries 9–13). Although the reaction time using 15 or 20 mg of the catalyst loadings is slightly less than compared to 10 mg loading in EtOH under reflux conditions, no noticeable change in efficiency was seen. For this reason, the optimal amount of catalyst was chosen to be 10 mg. Furthermore, by using the introduced catalyst components separately in the model reaction, it can be concluded that the prepared nanocatalyst shows better results in proceeding the three-component synthesis of imidazole derivatives. Hence, 10 mg of catalyst Fe_3_O_4_/SiO_2_-TMA-Me (**1**) loading in EtOH under reflux conditions was selected as the optimal conditions for the next experiments.

The optimized conditions were developed to different aromatic aldehydes affording other imidazole derivatives. The results are summarized in Table 2. Noticeably, the desired products **6a–m** were obtained in high to excellent yields. The obtained results obviously confirm the applicable catalytic activity of the Fe_3_O_4_/SiO_2_-TMA-Mel nanomaterial (**1**) to promote the three-component condensation of a wide range of aldehydes with benzil or benzoin and ammonium acetate (Supplementary Figure S1).

**Table 2:** Scope of the three-component synthesis of imidazole derivatives catalyzed by the Fe_3_O_4_/SiO_2_-TMA-Me (**1**) nanocomposite^a^.


Entry	Aldehyde (4)	Product 6	Time (min) “Benzoin”	Time (min) “Benzil”	Yield (%) “Benzoin”	Yield (%) “Benzil”	Observed Melting Point ( °C)
**1**	4-Chlorobenzaldehyde (**4a**)	2-(4-Chlorophenyl)-4,5-diphenyl-1*H*-imidazole (**6a**)	35	30	95	98	258–259^35^
**2**	Benzaldehyde (**4b**)	2,4,5-Triphenyl-1*H*-imidazole (**6b**)	65	63	82	86	270–272^36^
**3**	2-Chlorobenzaldehyde (**4c**)	2-(2-Chlorophenyl)-4,5-diphenyl-1*H*-imidazole (**6c**)	45	35	89	93	197–199^37^
**4**	4-Methylbenzaldehyde (**4d**)	4,5-Diphenyl-2-(p-tolyl)-1*H*-imidazole (**6d**)	75	73	80	84	188–190^38^
**5**	4-Methoxybenzaldehyde (**4e**)	2-(4-Methoxyphenyl)-4,5-diphenyl-1*H*-imidazole (**6e**)	65	55	89	92	228–229^39^
**6**	2,4-Dichlorobenzaldehyde (**4f.**)	2-(2,4-Dichlorophenyl)-4,5-diphenyl-1*H*-imidazole (**6f**)	55	45	80	84	178^40^
**7**	4-Nitrobenzaldehyde (**4 g**)	2-(4-Nitrophenyl)-4,5-diphenyl-1*H*-imidazole (**6g**)	45	38	88	92	236–238^41^
**8**	4-(Dimethylamino)benzaldehyde (**4 h**)	4-(4,5-Diphenyl-1*H*-imidazol-2-yl)-*N*,*N*-dimethylaniline (**6h**)	100	90	70	83	228–229^42^
**9**	2-Thiophenecarboxaldehyde (**4i**)	4,5-Diphenyl-2-(thiophen-2-yl)-1*H*-imidazole (**6i**)	90	83	70	79	263–265^39^
**10**	Furan-2-carbaldehyde (**4j**)	2-(Furan-2-yl)-4,5-diphenyl-1*H*-imidazole (**6j**)	85	80	80	85	200–202^39^
**11**	Pyrrole-2-carboxaldehyde (**4 k**)	4,5-Diphenyl-2-(1*H*-pyrrol-2-yl)-1*H*-imidazole (**6k**)	100	90	68	75	200–203^43^
**12**	2-Pyridinecarboxaldehyde (**4 l**)	4,5-Diphenyl-2-(2-pyridyl)-1*H*-imidazole **(6l)**	120	95	70	78	190–192^44^
**13**	3-Nitrobenzaldehyde (**4 m**)	2-(3-Nitrophenyl)-4,5-diphenyl-1*H*-imidazole (**6m**)	50	40	85	90	300–301^45^

^*a*^Reaction conditions: benzil or benzoin (**2** or **3**, 1 mmol), aldehyde derivatives (**4a–m**, 1 mmol) and ammonium acetate (**5**, 2.5 mmol) in the presence of 10 mg of Fe_3_O_4_/SiO_2_-TMA-Me (**1**) in EtOH under reflux conditions.

**Figure 9:**
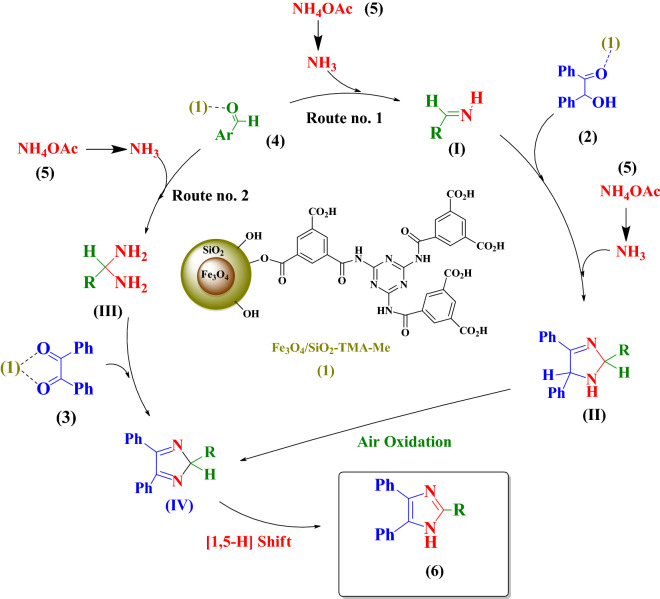
Plausible mechanism for the imidazole derivatives synthesis catalyzed by the nanocomposite Fe_3_O_4_/SiO_2_-TMA-Me nanocomposite (**1**).

According to above results presented in Table 2, the following mechanism can be proposed for the synthesis of imidazole derivatives **6** by starting from benzil (**2**) or benzoin (**3**) catalyzed by nanocatalyst **1** (Fig. 9). First, Fe_3_O_4_/SiO_2_-TMA-Me solid acid (**1**) activates the carbonyl functional group of aldehydes (**4**) followed by addition of ammonia source (ammonium acetate **5**) and forming imine intermediate (**I**) from route no.1 or aminal intermediate (**III**) from route no. 2. Subsequent addition of benzoin (**2**) and ammonia to the imine intermediate (**I**), followed by cyclization, air oxidation through intermediate (**II**) in route no. 1 or benzil (**3**) affords intermediate (**IV**). Finally, [1,5–H] shift of the intermediate (**IV**) affords the desired imidazole derivatives **6**.

**Figure 10:**
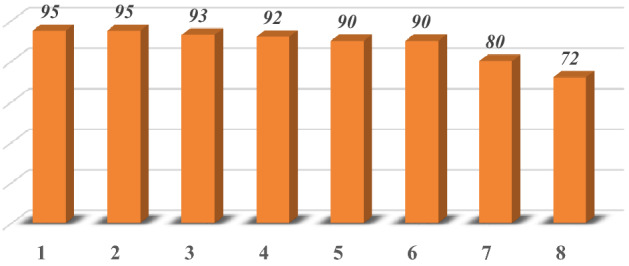
Reusability of the Fe_3_O_4_/SiO_2_-TMA-Me heterogeneous catalyst (**1**) in the model reaction to afford **6a**.

**Figure 11:**
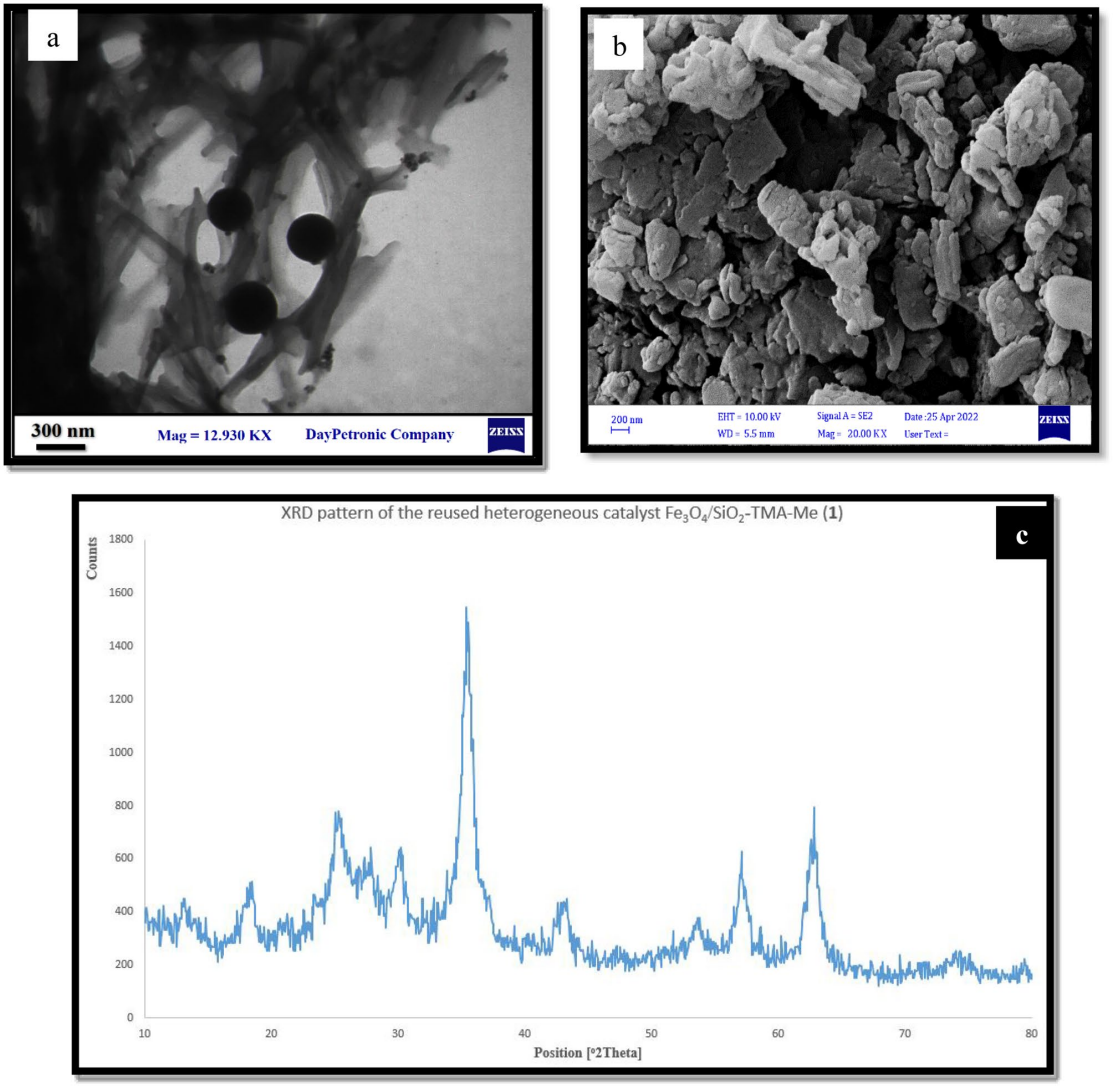
(**a**) TEM and (**b**) FESEM images and (**c**) XRD pattern of the reused Fe_3_O_4_/SiO_2_-TMA-Me heterogeneous acid (**1**) in the model reaction to afford **6a**.

One of the important advantages of Fe_3_O_4_/SiO_2_-TMA-Me nanomaterial (**1**) is that it can be magnetically separated from the reaction mixture after each run, collected, washed using acetone and n-hexane, respectively, and then reused in the subsequent model reactions. The model reaction was performed using the recycled catalyst for several times. As a result, a slight decrease in the catalytic efficiency was observed after the seventh run (Fig. 10). TEM and FESEM images as well as XRD pattern of the reused heterogeneous catalyst Fe_3_O_4_/SiO_2_-TMA-Me (**1**) have been presented in Fig. 11, which show excellent stability of the catalyst **1** under optimized reaction conditions.

To evaluate the efficiency of Fe_3_O_4_/SiO_2_-TMA-Me acidic catalyst (**1**), a comparison has been made with the previously reported methods for the synthesis of imidazole derivative **6a**. As shown in Table 3, the prepared nanocatalyst can compete with the  similar systems in terms of catalyst loading, reaction conditions, and catalyst reusability times.

**Table 3:** Comparison of the results obtained for the synthesis of imidazole **6a** using benzoin (**3**) in the presence of Fe_3_O_4_/SiO_2_-TMA-Me solid acid (**1**) and other catalysts.

Entry	Catalyst	Reaction conditions	Catalyst loading	Time (min)	Yield (%)	No. of reuse	Reference
1	Brønsted acidic ionic liquid, *N*-methyl-2-pyrrolidonium hydrogen sulfate	Solvent-free, 100 °C	80 mg	120	95	7	^46^
2	Ytterbium perfluorooctanesulfonate	HOAc, perfluorodecalin; 80 °C	6.7 mg	360	83	–	^47^
3	Zirconium chloride	CH_3_CN, r.t	46.7 mg	600	93^a^	–	^48^
4	Ferric(III) nitrate supported on kieselguhr (Fe(NO_3_)_3_-Kie)	Solvent-free, 120 °C	1.6 mmol%	60	89^a^	3	^49^
5	Pyromellitic diamide–diacid bridged mesoporous organosilica	EtOH/reflux	15 mg	40	98	6	^39^
6	Nano-Ceramic tile waste-SO_3_H	Solvent-free, 100 °C	20 mol%	20	95^a^	7	^40^
7	Fe_3_O_4_/SiO_2_ decorated trimesic acid-melamine nanocomposite (Fe_3_O_4_/SiO_2_-TMA-Me, **1**)	Reflux/EtOH	10 mg	35	95	7	This work

### Experimental section

#### General information

All consumable chemicals were obtained from Merck or Aldrich chemical companies. The XRD pattern was collected by a TW 1800 diffractometer with Cu Ka radiation (λ = 1.54050 Å). The FESEM images were observed by FESEM TESCAN-MIRA3. TEM images were taken using a JEOL JEM-2100F microscope (operated at 300 kV). The analytical thin layer chromatography (TLC) experiments were performed using Merck 0.2 mm silica gel 60F-254Al-plates and n-hexane: EtOAc, (3:1, v/v %) as eluent. All compounds are known and well characterized by melting point, FTIR, ^1^H NMR (500 MHz), and ^13^C NMR (125 MHz) spectroscopy on a Bruker DRX-500 Avance instrument in DMSO-*d*_*6*_ at ambient temperature.

#### General procedure for preparation of the Fe_3_O_4_/SiO_2_-TMA-Me (1)

The magnetic core/shell **(**Fe_3_O_4_/SiO_2_) material was prepared according to the reported methods in literature with a slight modification^50^.

The mixture of trimesic acid (TMA, 3 mmol), 1-hydroxybenzotriazole (HOBT, 3 mmol) and 1-ethyl-3-(3-dimethylaminopropyl)carbodiimide (EDCI, 3 mmol) was stirred in deionized water/acetonitrile (1:1, 50 mL) for 30 min, then 1 mmol of melamine was added and the obtained mixture was stirred for 24 h at room temperature. After this time, 0.3 g prepared Fe_3_O_4_/SiO_2_ was mildly added and stirred for 24 h to afford the final precipitate. Afterward, the obtained solid was collected with an external magnet, washed several times using distilled water and EtOH (96%) and then dried at 45 °C for 3 h.

#### General procedure for the synthesis of imidazole derivatives 6a–m catalyzed by the Fe_3_O_4_/SiO_2_-TMA-Me (1)

In a round-bottomed flask, benzoin (**2**, 1.0 mmol) or benzil (**3**, 1.0 mmol), aldehyde (**4**, 1.0 mmol), ammonium acetate (**5**, 2.5 mmol) and Fe_3_O_4_/SiO_2_-TMA-Me (**1**, 10 mg) were mixed in EtOH (5.0 mL) and stirred at room temperature. The reaction mixture was stirred for the appropriate times reported in Table 2. After completion of the reaction, the catalyst **1** was separated by an external magnet. Afterwards, H_2_O was added drop wise into the solution until imidazole derivatives **6** were completely precipitated. The obtained mixture was filtered off and the precipitate were washed and then dried in an oven at 70 °C for 1 h. The recycled catalyst **1** was washed with acetone and n-hexane (1 mL), respectively and then dried at 50 °C for 2 h and stored for another run.

## Conclusion

The magnetic Fe_3_O_4_/SiO_2_ decorated trimesic acid-melamine pseudo-supramolecular (Fe_3_O_4_/SiO_2_-TMA-Me) nanocomaterial was prepared and properly characterized for the first time. The Fe_3_O_4_/SiO_2_-TMA-Me nanocomposite was used for the three-component condensation of benzil or benzoin, aldehydes, and ammonium acetate to afford the corresponding imidazole derivatives. Low catalyst loading, high to excellent yields of the desired products, easy and quick isolation of the products from the reaction mixture as well as reusability of the solid acidic pseudo-supramolecular nanocomposite with negligible loss of its activity are the main advantages of this method. In addition to the catalytic applications, other applications of this nanomaterial, as a pseudo-supramolecular structure, are ongoing in our laboratory and would be presented in due course.

## Supplementary Information

Supplementary Information.

## Data Availability

The datasets generated and/or analyzed during the current study would be available in the Science Data Bank repository after acceptance of the manuscript.
