# Supplementary material for: Fe3O4/SiO2 decorated trimesic acid-melamine nanocomposite: a reusable supramolecular organocatalyst for efficient multicomponent synthesis of imidazole derivatives
Source: Sci Rep. 2023 Jan 9;13:401. doi: 10.1038/s41598-023-27408-7 (PMC9829914; doi:10.1038/s41598-023-27408-7)
Supplement: Supplementary file 1 — Supplementary Information. [file 41598_2023_27408_MOESM1_ESM.docx]

**Fe_3_O_4_/SiO_2_ decorated trimesic acid-melamine nanocomposite: A reusable supramolecular organocatalyst for efficient multicomponent synthesis of imidazole derivatives**

Babak Fattahi and Mohammad G Dekamin*

Pharmaceutical and Heterocyclic Compounds Research Laboratory, Department of Chemistry, Iran University of Science and Technology, Tehran 16846‑13114, Iran.

*Email: mdekamin@iust.ac.ir

| **Page** | **Content** |
| --- | --- |
| S1 | Title page |
| S2 | General procedure for preparation of the Fe_3_O_4_/SiO_2_-TMA-Me nanocomposite (**1**) |
| S3 | Schematic preparation of Fe_3_O_4_/SiO_2_-TMA-Me (1) for the three-component condensation of benzil (**2**) or benzoin (**3**), aldehydes (**4**), ammonium acetate (**5**) to afford imidazole derivatives **6** |
| S4 | FTIR spectra of the Fe_3_O_4_/SiO_2_, melamine (Mel), trimesic acid (TMA), melamine-trimesic acid amide (Mel-TMA) and Fe_3_O_4_/SiO_2_-TMA-Me solid acid (**1**) |
| S5 | FESEM images of the Fe_3_O_4_/SiO_2_ decorated trimesic acid-melamine (Fe_3_O_4_/SiO_2_-TMA-Me) nanocomposite (**1**) |
| S6 | TEM images of the Fe_3_O_4_/SiO_2_ decorated trimesic acid-melamine (Fe_3_O_4_/SiO_2_-TMA-Me) nanocomposite (**1**) in 1.0 µm and 300 nm scales |
| S7 | Energy dispersive spectroscopy (EDX) pattern of the Fe_3_O_4_/SiO_2_ decorated trimesic acid-melamine (Fe_3_O_4_/SiO_2_-TMA-Me) nanocomposite (**1**) |
| S7 | XRD patterns of of the Fe_3_O_4_/SiO_2_ decorated trimesic acid-melamine (Fe_3_O_4_/SiO_2_-TMA-Me) nanocomposite (**1**) |
| S8 | General procedure for the synthesis of imidazole **6a–m** catalyzed by the Fe_3_O_4_/SiO_2_-TMA-Me) nanocomposite (**1**) |
| S8 | Spectral characterization of compounds **6a**, **6c** and and **6d** |

**General procedure for preparation of the Fe_3_O_4_/SiO_2_-TMA-Me (1)**

The magnetic core/shell **(**Fe_3_O_4_/SiO_2_) material were prepared according to the reported methods in literature with a slight modification [1].

The mixture of trimesicacid (3 mmol), HOBT (3 mmol) and EDCI (3 mmol) was stirred in 50 mL deionized water/acetonitrile (1:1) for 30 min, then 1 mmol of melamine was added and the obtained mixture was stirred for 24 h at room temperature. After this time, 0.3 g prepared Fe_3_O_4_/SiO_2_ was mildly added and stirred for 24 h to afford the final precipitate. Afterward, the obtained solid was collected with magnet, washed several times and dried at 45 °C.

[1] Ishani, M., Dekamin, M. G. & Alirezvani, Z. Superparamagnetic silica core-shell hybrid attached to graphene oxide as a promising recoverable catalyst for expeditious synthesis of TMS-protected cyanohydrins. *Journal of Colloid and Interface Science* **521**, 232-241, doi:<https://doi.org/10.1016/j.jcis.2018.02.060> (2018).

**Fig. S1**. Schematic preparation of Fe_3_O_4_/SiO_2_-TMA-Me (1) for the three-component condensation of benzil (**2**) or benzoin (**3**), aldehydes (**4**), ammonium acetate (**5**) to afford imidazole derivatives **6**.

**Fig. S2.** FTIR spectra of the Fe_3_O_4_/SiO_2_, melamine (Mel), trimesic acid (TMA), melamine-trimesic acid amide (Mel-TMA) and Fe_3_O_4_/SiO_2_-TMA-Me solid acid (**1**).


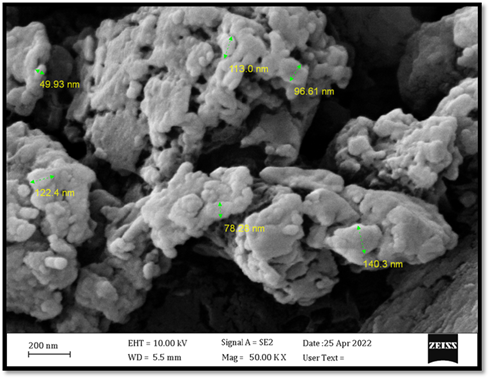

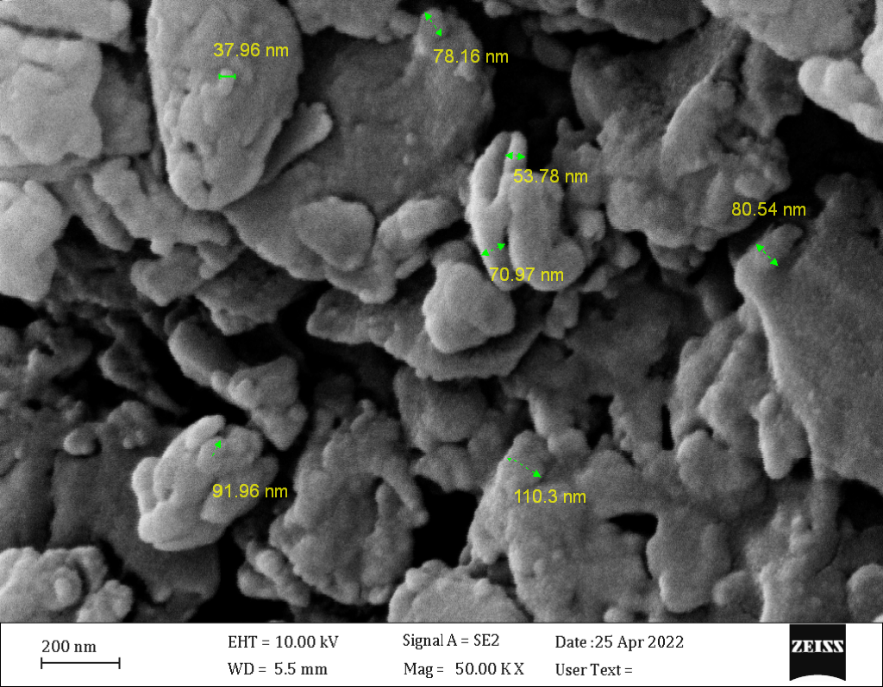


**Fig. S3.** FESEM images of the Fe_3_O_4_/SiO_2_ decorated trimesic acid-melamine (Fe_3_O_4_/SiO_2_-TMA-Me) nanocomposite (**1**).


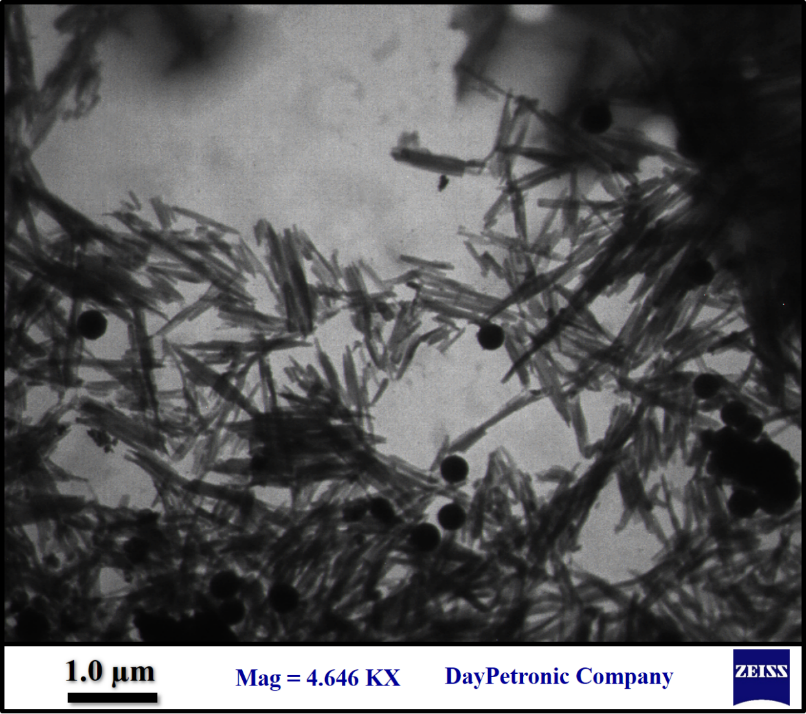


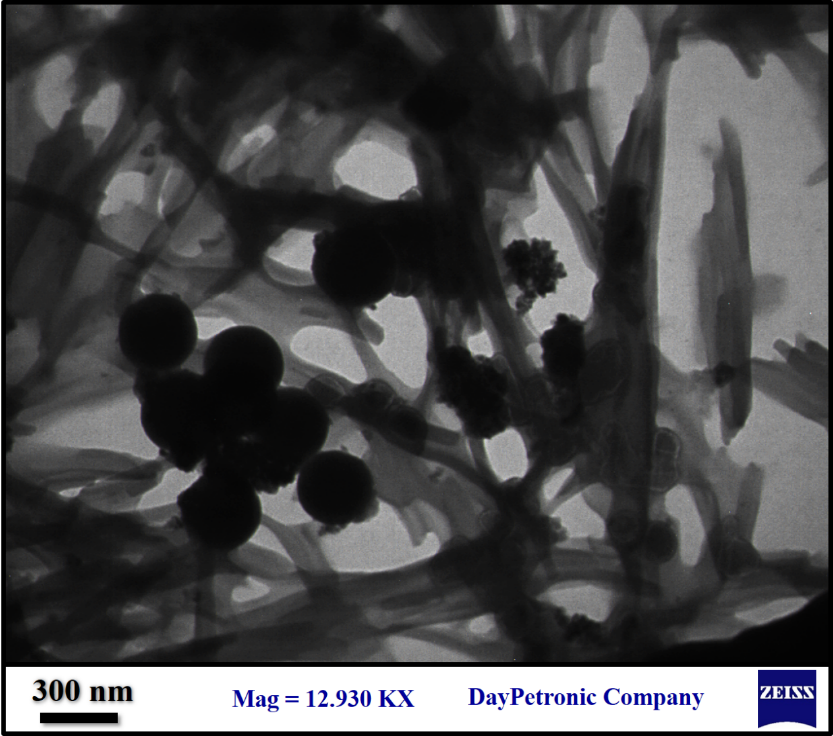


**Fig. S4**. TEM images of the Fe_3_O_4_/SiO_2_ decorated trimesic acid-melamine (Fe_3_O_4_/SiO_2_-TMA-Me) nanocomposite (**1**) in 1.0 µm and 300 nm scales.


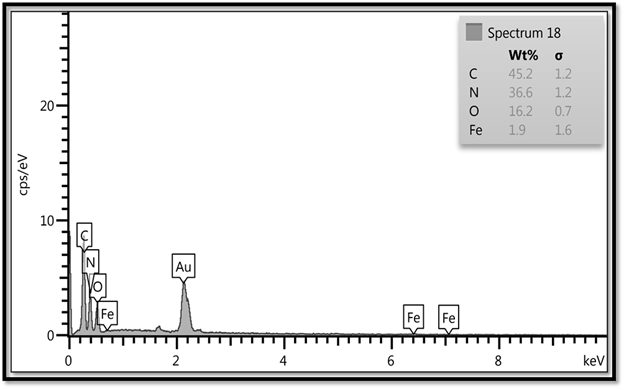


**Fig. S5.** Energy dispersive spectroscopy (EDX) pattern of the Fe_3_O_4_/SiO_2_ decorated trimesic acid-melamine (Fe_3_O_4_/SiO_2_-TMA-Me) nanocomposite (**1**).


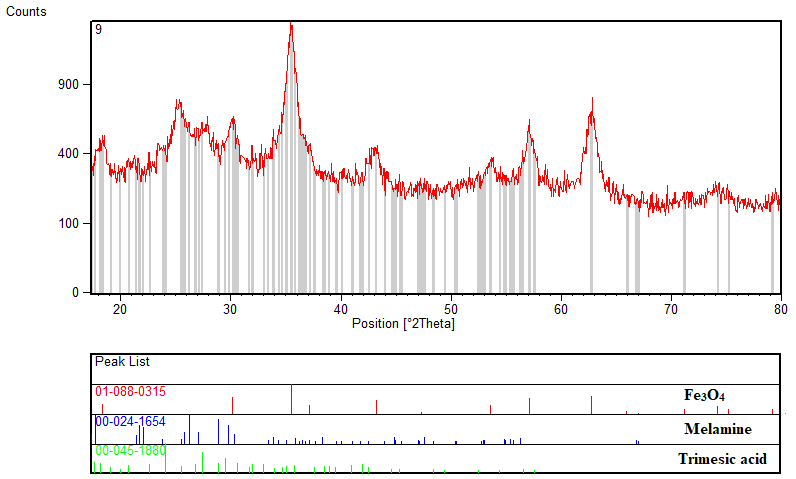


**Fig. S6.** XRD patterns of of the Fe_3_O_4_/SiO_2_ decorated trimesic acid-melamine (Fe_3_O_4_/SiO_2_-TMA-Me) nanocomposite (**1**).

**General procedure for the synthesis of imidazole derivatives 6a–m catalyzed by the Fe_3_O_4_/SiO_2_-TMA-Me (1)**

In a round-bottomed flask, benzoin (**2**, 1.0 mmol) or benzil (**3**, 1.0 mmol), various aldehydes (**4**, 1.0 mmol), ammonium acetate (**5**, 2.5 mmol) and Fe_3_O_4_/SiO_2_-TMA-Me (**1**, 10 mg) were mixed in EtOH (5.0 mL) and stirred at room temperature. The reaction mixture was stirred for the appropriate times reported in Table 2. After completion of the reaction, the catalyst **1** was separated by an external magnet. Afterwards, H_2_O was added drop wise into the solution until imidazole derivatives **6** were completely precipitated. The obtained mixture was filtered off and the precipitate were washed and then dried in an oven at 70 °C for 1 h. The recycled catalyst **1** was washed with acetone and n-hexane (1 mL), respectively and then dried at 50 °C for 2 h and stored for another run.

**Spectral characterization of compounds 6a, 6c and and 6d:**

2-(4-chlorophenyl)-4,5-diphenyl-1*H*-imidazole (**6a**):

M.P = 258-259°C; FTIR (KBr, cm^–1^): 3434, 3032, 1599, 1493, 1420, 1172, 923, 760, 690; (500 MHz, DMSO-*d*_6_): δ (ppm) =7.32 – 7.87 (m, 14H), 10.31 (br, NH); ^13^C NMR (100 MHz, DMSO-d6): δ (ppm) = 127.45, 128.80, 128.05, 128.77, 129.09, 129.57, 129.67, 12.88, 130.51, 130.95, 132.40, 143.24.


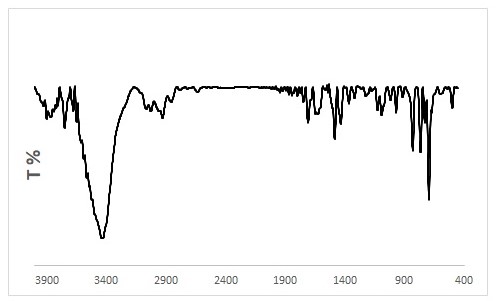


**Fig. S7.** FTIR spectrum of 2-(4-chlorophenyl)-4,5-diphenyl-1*H*-imidazole (**6a**).


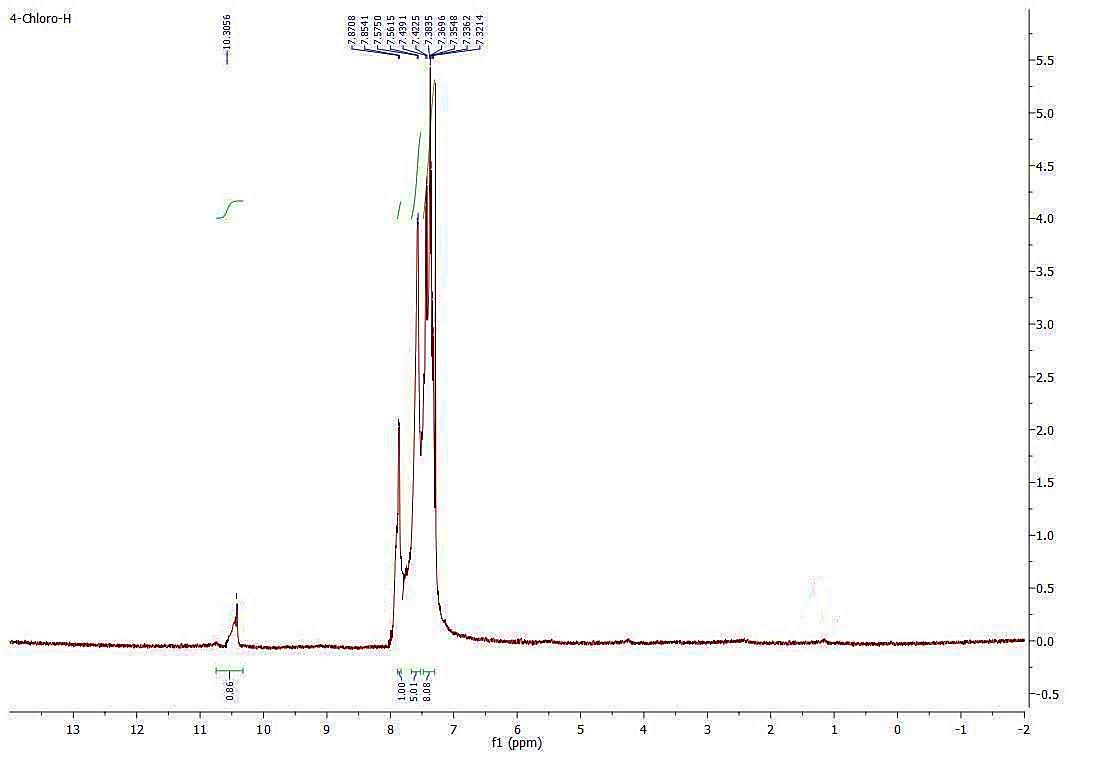


**Fig. S8.** ^1^H NMR spectrum of 2-(4-chlorophenyl)-4,5-diphenyl-1*H*-imidazole (**6a**).


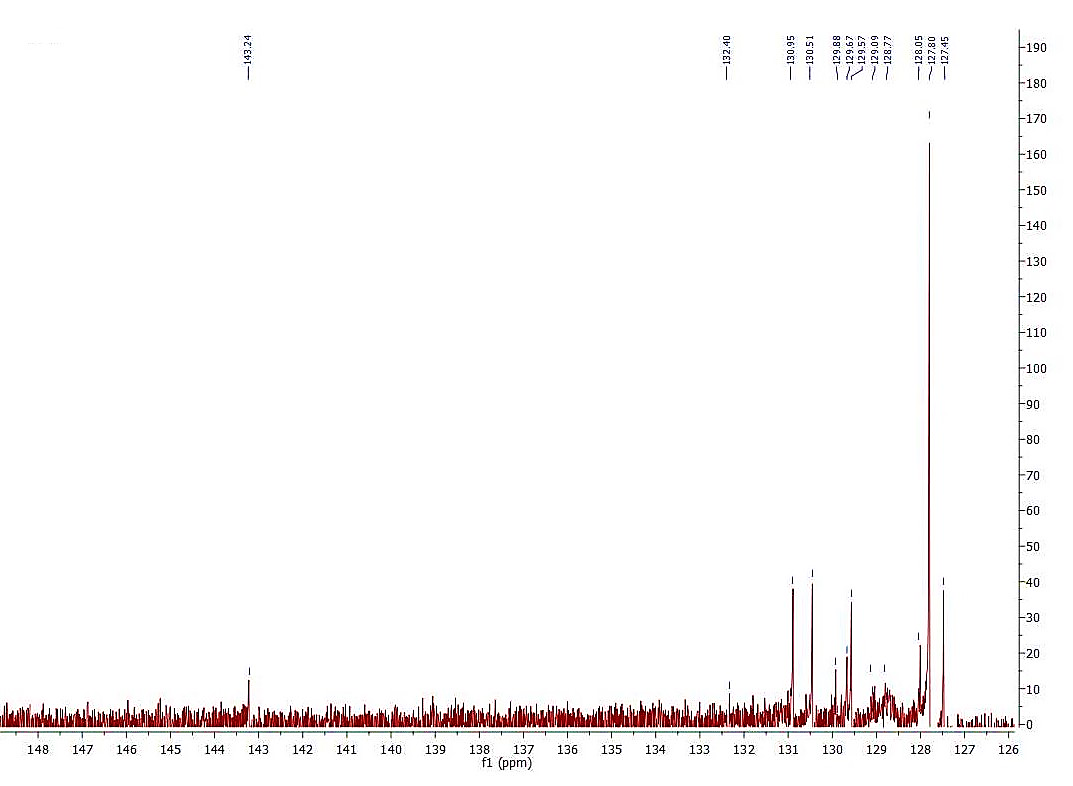


**Fig. S9.** ^13^C NMR of 2-(4-chlorophenyl)-4,5-diphenyl-1*H*-imidazole (**6a**).

2-(2-chlorophenyl)-4,5-diphenyl-1*H*-imidazole (**6c**):

M.P = 197-199°C; FTIR (KBr, cm^–1^): 3422, 3030, 1603, 1493, 1440, 1072, 823, 730, 696; (500 MHz, DMSO-*d*_6_): δ (ppm) = 7.23–7.800 (m, 14H), 12.65 (br, NH).


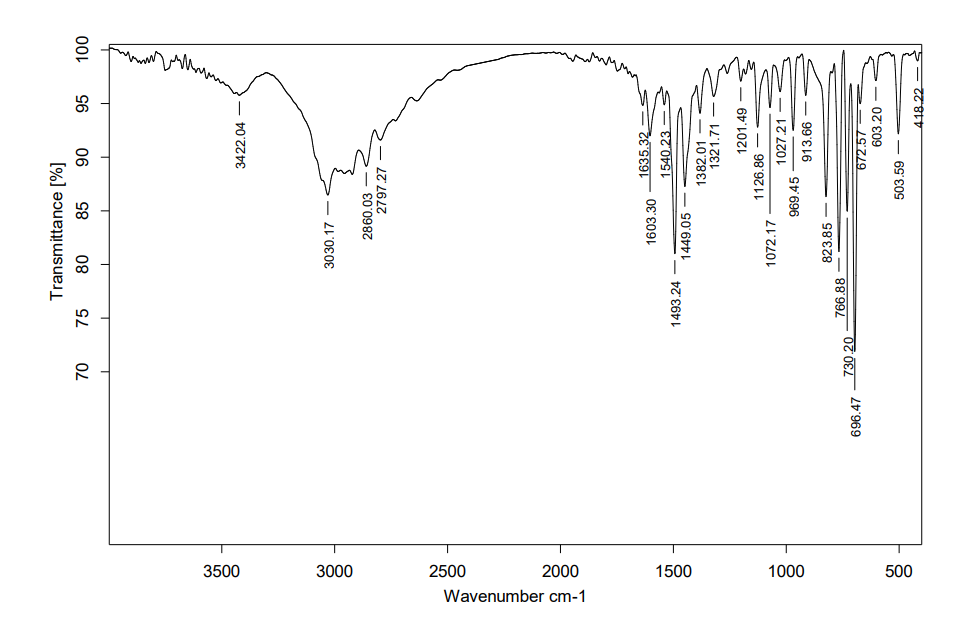


**Fig. S10.** FTIR spectrum of 2-(2-chlorophenyl)-4,5-diphenyl-1*H*-imidazole (**6c**).


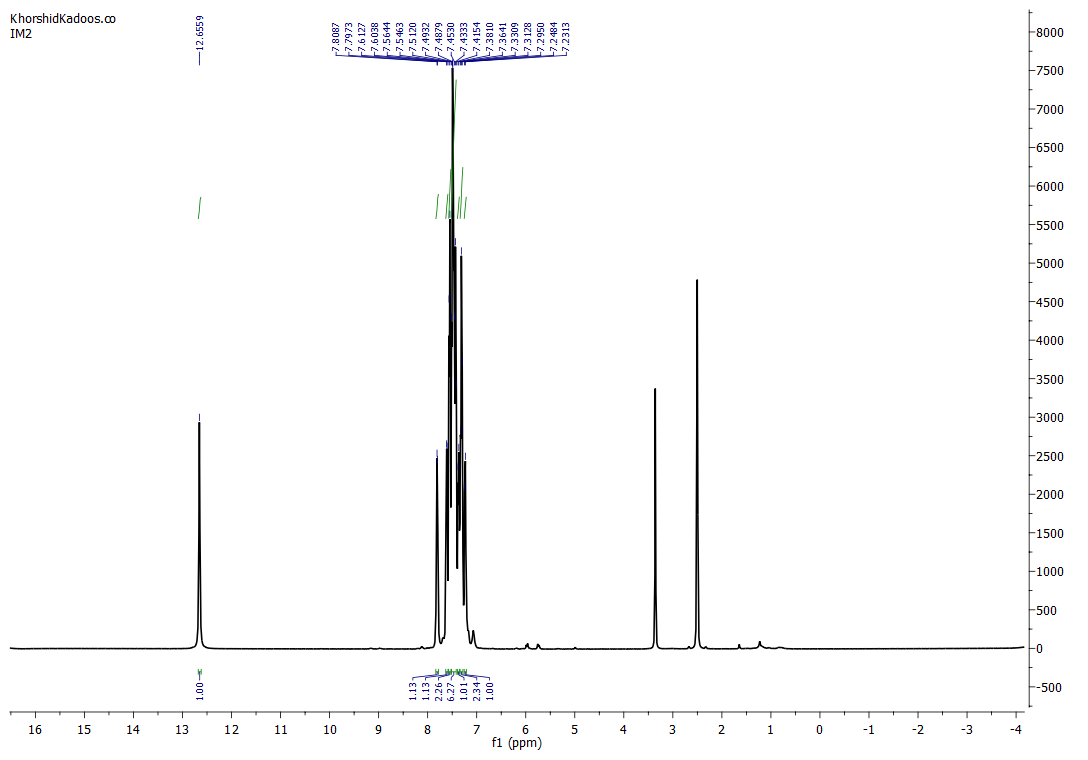


**Fig. S11.** ^1^H NMR spectrum of 2-(2-chlorophenyl)-4,5-diphenyl-1*H*-imidazole (**6c**).

4,5-diphenyl-2-(p-tolyl)-1*H*-imidazole (**6d**):

M.P = 188-190 °C; FTIR (KBr, cm^–1^): 3444, 3025, 1602, 1476, 1445, 1392, 1063, 762, 694; ^1^H NMR (500 MHz, DMSO-*d*_6_): δ (ppm) = 2.35 (s, Me), 7.20–7.99 (m, 14H), 12.6 (br, NH).


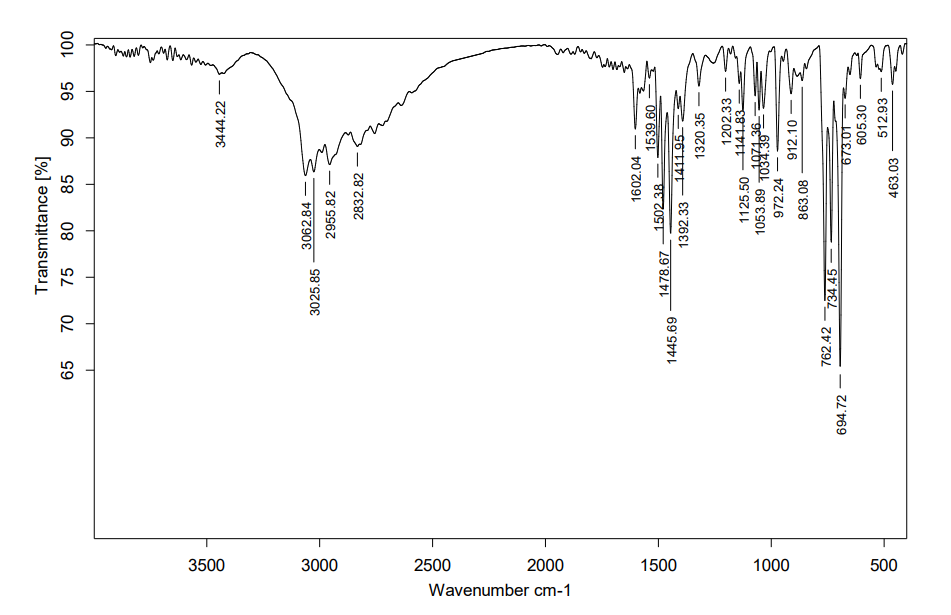


**Fig. S12.** FTIR spectrum of 4,5-diphenyl-2-(*p*-tolyl)-1*H*-imidazole (**6d**).


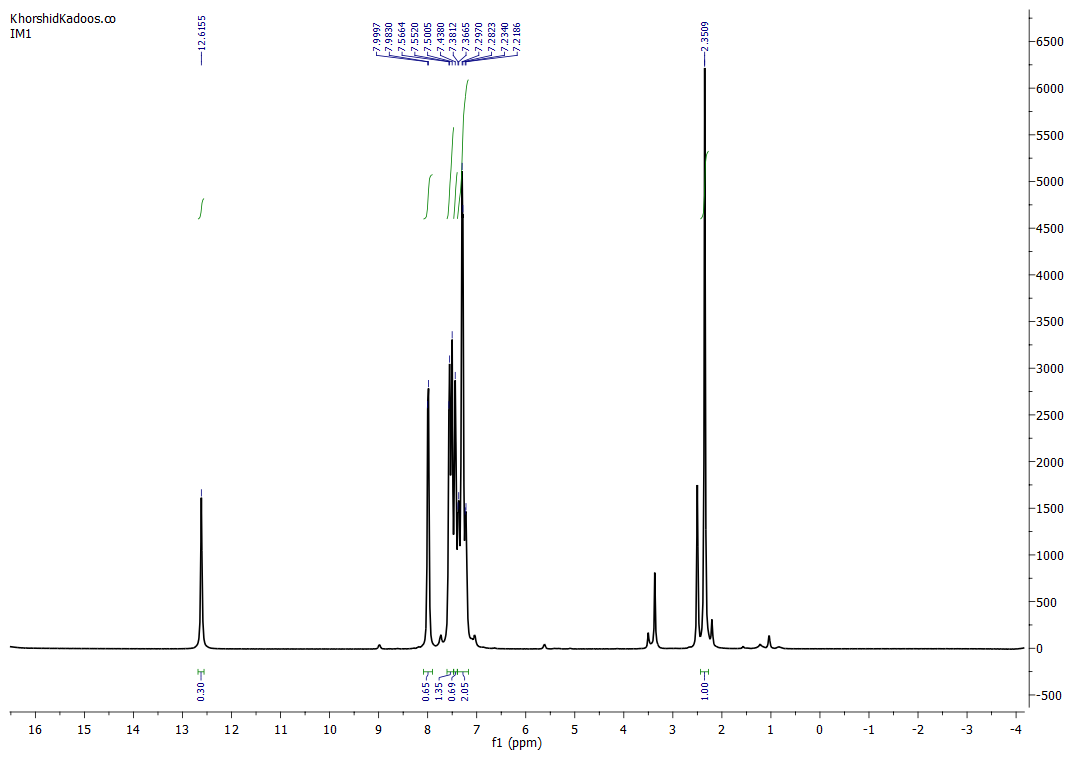


**Fig. S13.** ^1^H NMR spectrum of 4,5-diphenyl-2-(*p*-tolyl)-1*H*-imidazole (**6d**).
